# Data presenting the synthesis of three novel stimuli responsive hyperbranched polymers synthesised via RAFT polymerisation and the bio conjugation of folic acid

**DOI:** 10.1016/j.dib.2019.104861

**Published:** 2019-11-21

**Authors:** Chester Blackburn, Hongyun Tai, Martina Salerno, Xi Wang, Chandra Senan, Ian Ratcliffe, Edgar Hartsuiker, Wenxin Wang

**Affiliations:** a School of Natural Sciences, Bangor University, Deiniol Road, Bangor, Gwynedd, LL57 2UW, UK; b NWCR Institute, School of Medical Sciences, Bangor University, Deiniol Road, Bangor, Gwynedd, LL57 2UW, UK; c Charles Institute of Dermatology, School of Medicine, University College Dublin, Dublin 4, Ireland; d Centre for Water Soluble Polymers, Wrexham Glyndwr University, Wrexham, LL11 2AW, Wales, UK

**Keywords:** Hyperbranched polymers, pH responsive, RAFT polymerisation, Targeted delivery

## Abstract

The data presented in this manuscript presents the characterisation spectra of three hyperbranched polymers as discussed in the paper “Folic Acid and Rhodamine Labelled pH Responsive Hyperbranched Polymers: synthesis, characterisation and cell uptake studies” [1]. Characterisation of polymers was performed via ^1^H Nuclear Magnetic Resonance (^1^H NMR) and Size Exclusion Chromatography (SEC). pH responsive characteristics were observed via Dynamic Light Scattering (DLS). The data for characterisation of folate conjugated hyperbranched polymer is presented as ^1^H NMR, Ultra Violet Visible (UV-VIS) spectra and DLS measurements. Further data is presented detailing the experiments for the synthesis of monomers 2-propyl acrylic acid (PAA) and disulfide diacrylate (DSDA), with the full synthesis of folic acid-poly (ethylene glycol) (PEG) linker, rhodamine B ethylenediamine linker and bioconjugation reactions also detailed.

**Table:** Specifications Table

Subject	Materials Chemistry
Specific subject area	Hyperbranched stimuli responsive polymers for targeted drug delivery synthesised via RAFT polymerisation
Type of data	Table Graph Schemes
How data were acquired	The data was acquired from the following sources: ^1^H NMR data – Bruker Ultrashield 400Mhz for 16 scans analysed on MestReNova V6. For Fig. 9^1^H NMR data was acquired from a Bruker Ultrashield 500Mhz machine for 512 scans, analysed on MestReNova V6. SEC data – Agilent 1260 Infinity machine equipped with Polargel-M organic column using DMF as the eluent solvent system (additive 0.1% w/v LiBr) UV-VIS data - Perkin Elmer Lambda 35 UV/Vis spectrophotometer in 10 mm Quartz cells in DMF at ambient temperature, analysed with Microsoft Excel DLS particle sizing data - Malvern Zetasizer 1000 Hsa at 25 °C with 90° forward scatter optics and a 633 nm laster, analysed using PCS software V1.61.
Data format	Raw Analysed
Parameters for data collection	Data was collected for characterisation purposes. After purification of polymers via precipitation, ^1^H NMR data was collected, alongside SEC data. After bio-conjugation, dialysis was performed and DLS, UV-VIS and ^1^H NMR data was acquired.
Description of data collection	Data was collected via the raw output files from the respective hardware. ^1^H NMR was recorded as.fid files. UV-VIS data was collected as.csv files. DLS data was obtained as.SZ2 files.
Data source location	Bangor University, Bangor, Gwynedd, United Kingdom Wrexham Glyndwr University, Wrexham, United Kingdom University College Dublin, Dublin, Ireland
Data accessibility	With the article
Related research article	Chester Blackburn, Hongyun Tai, Martina Salerno, Xi Wang, Edgar Hartsuiker and Wenxin Wang Folic Acid and Rhodamine Labelled pH Responsive Hyperbranched Polymers: synthesis, characterisation and cell uptake studies, *European Polymer Journal*, DOI: na

**Table:** 

**Value of the Data** • Theses data presents the ^1^H NMR spectra of synthesised hyperbranched polymers, with dynamic light scattering (DLS) within different pH media of the polymer HBP4060, in order to assess particle size. UV spectra obtained display conjugation of folic acid onto this polymer, alongside further DLS data to corroboration. • These data can be used by researchers in the field of polymer drug delivery to evaluate the suitability of these structures for their purposes. The data will allow researchers to make modifications to this polymer and make comparisons. • By using the ^1^H NMR data provided, researchers can be assisted in elucidating peaks in the spectra. Researchers can also benefit from UV data for peak identification relating to the RAFT agent used and folic acid peaks. DLS data allows researchers to better understand the behaviour of this structure within an acidic environment.

## 1 Data

**Fig. 1:**
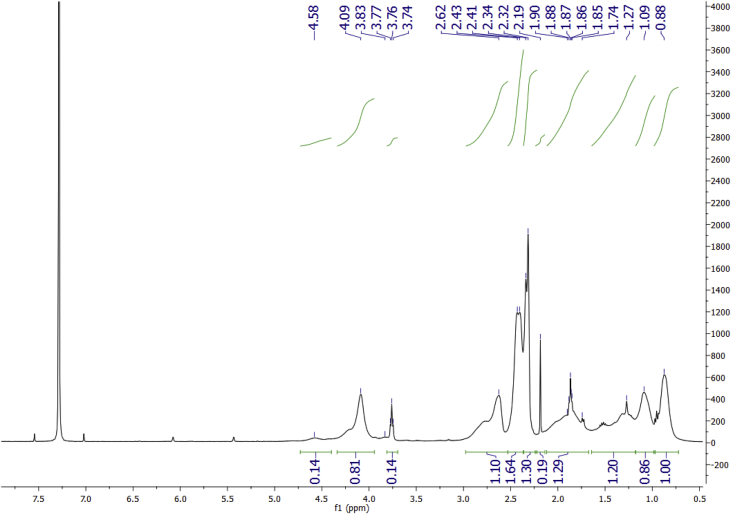
^1^H NMR of synthesised HBP5050.

**Fig. 2:**
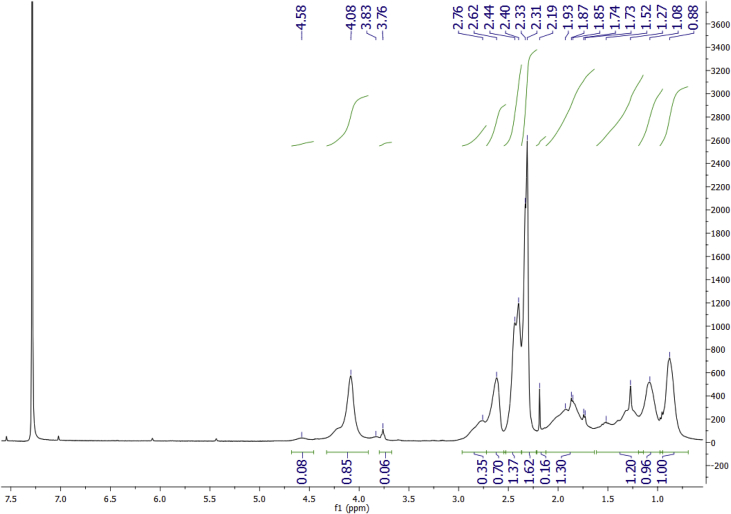
^1^H NMR of synthesised HBP4060.

**Fig. 3:**
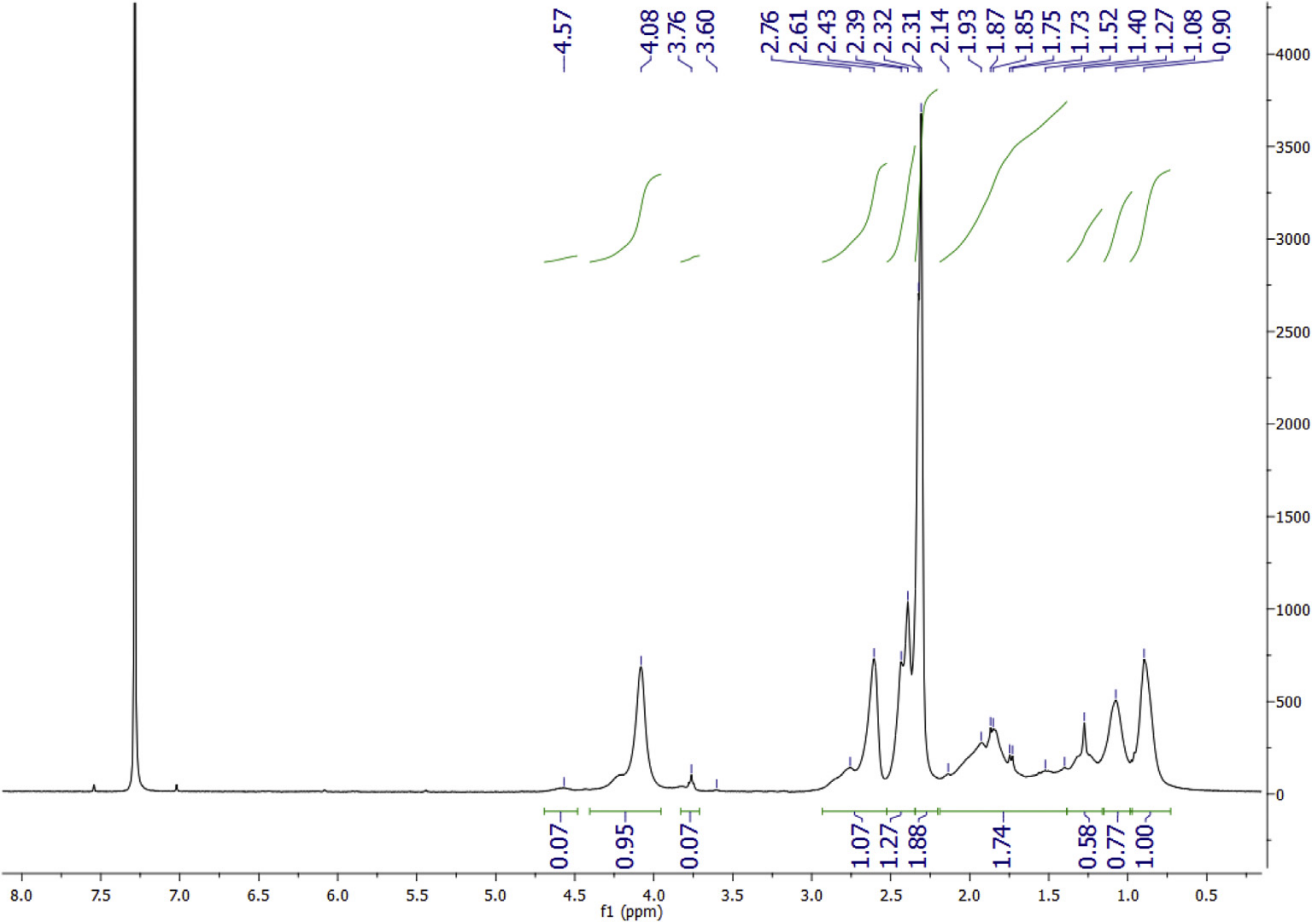
^1^H NMR of synthesised HBP3070.

**Fig. 4:**
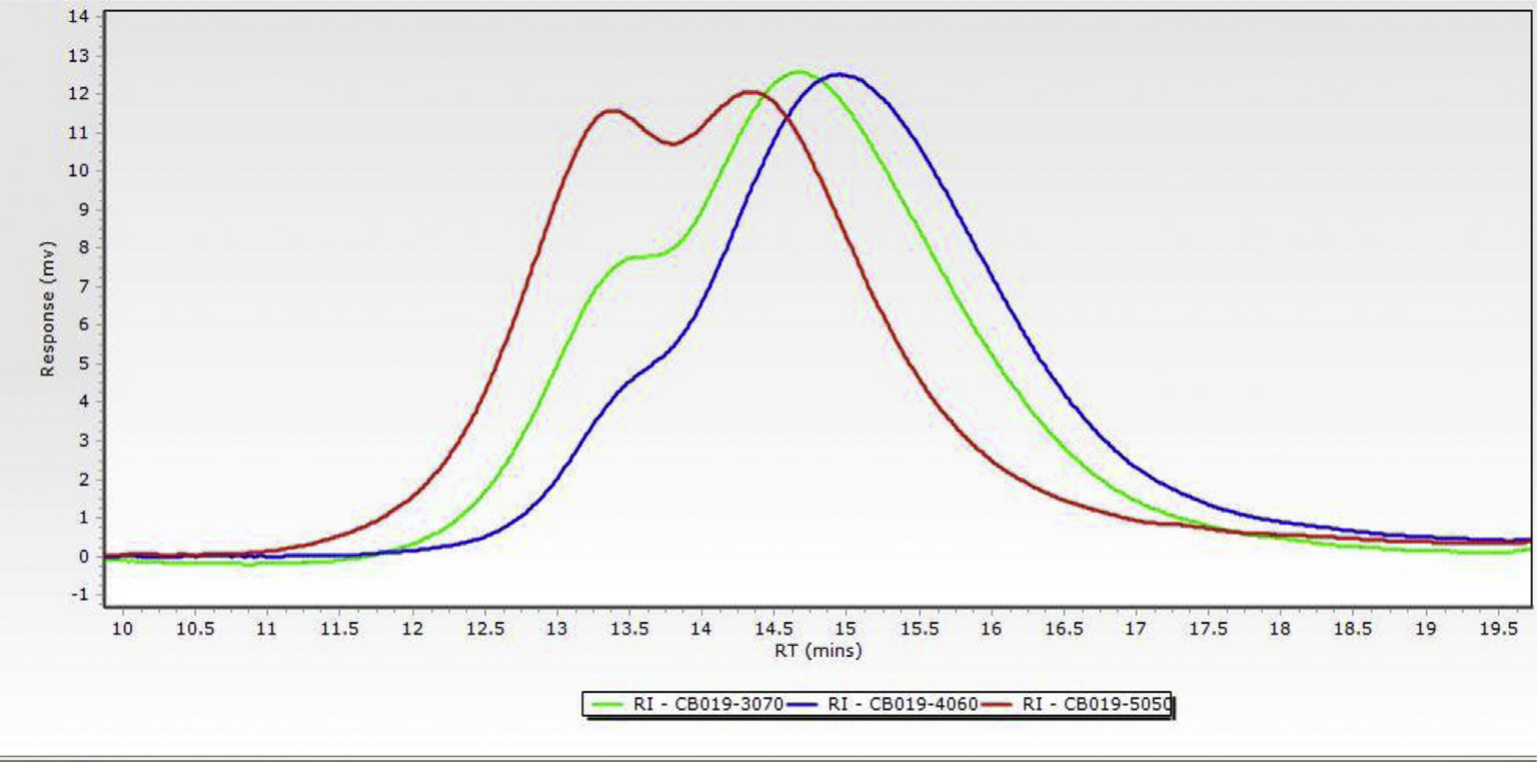
SEC traces for HBPs HBP3070 (Green) HBP4060 (Blue) and HBP5050 (Red).

**Fig. 5:**
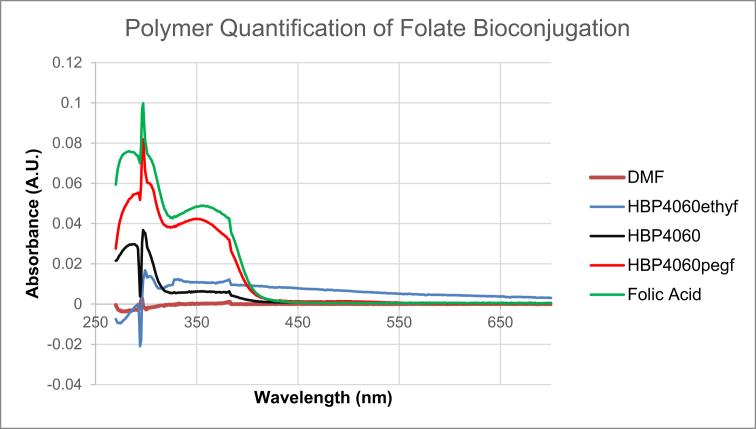
UV spectra for folic acid, HBP4060 and HBP4060 folate conjugates.

**Fig. 6:**
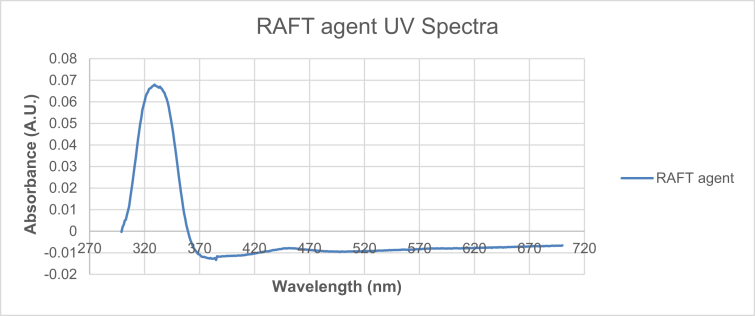
UV spectra for RAFT agent.

**Fig. 7:**
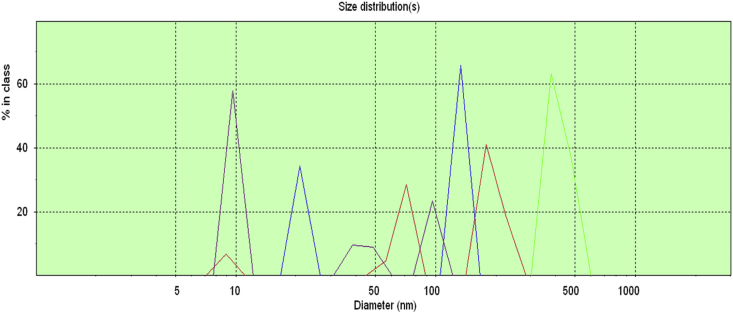
PCS peak output as a function of intensity for HBP4060 in differing media.

**Fig. 8:**
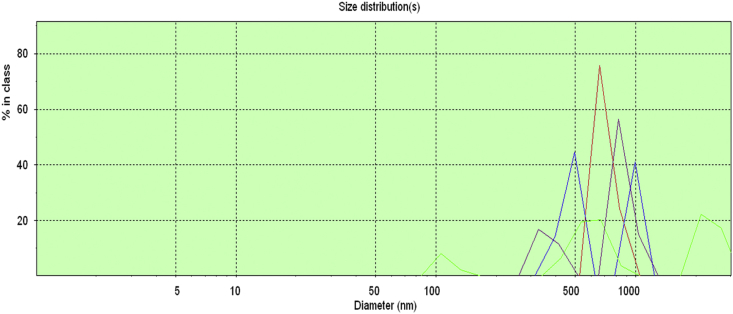
PCS peak output as a function of intensity for HBP4060_pegf_ in differing media.

**Fig. 9:**
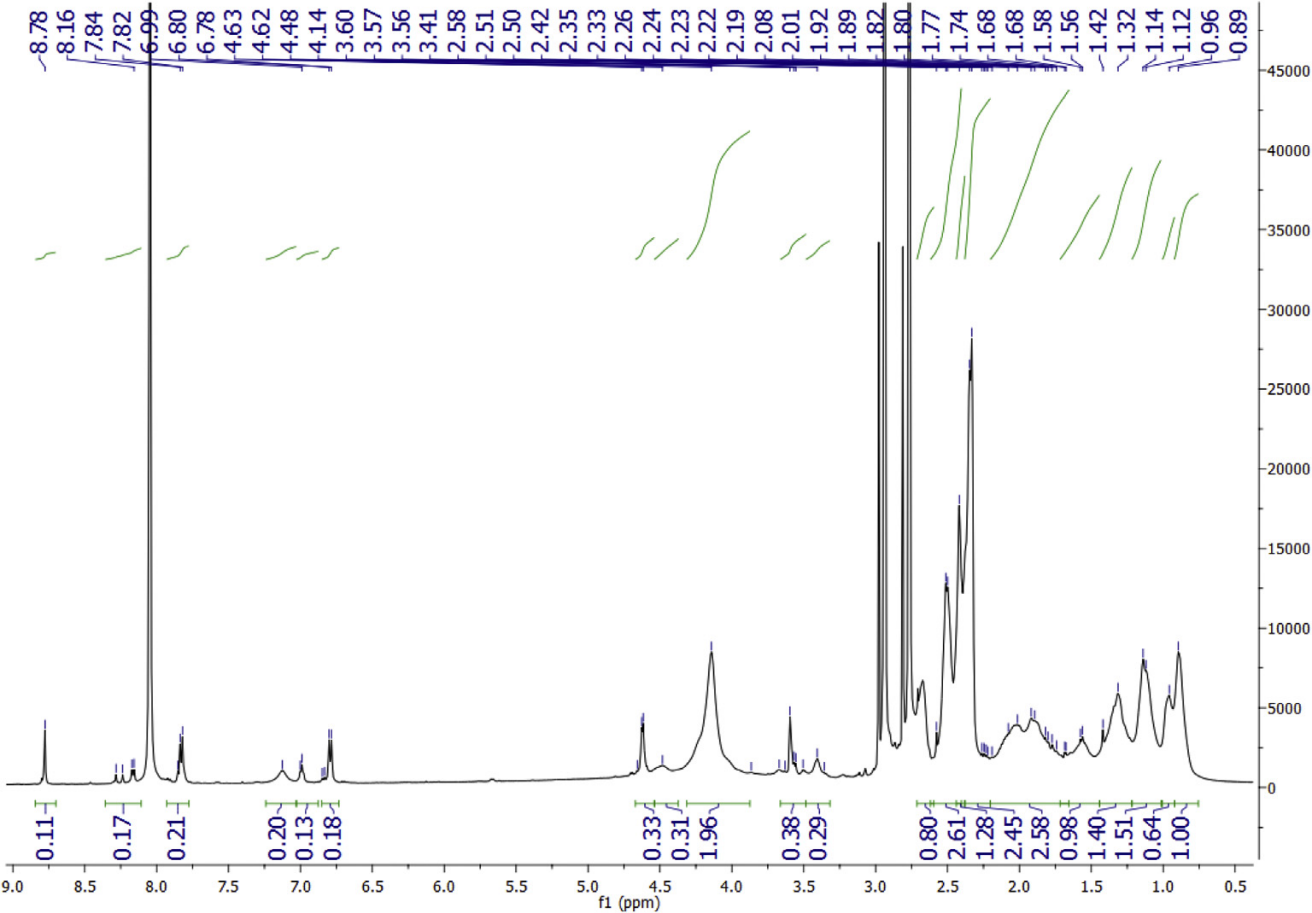
^1^H NMR of synthesised HBP4060_pegf_.

**Scheme 1:**
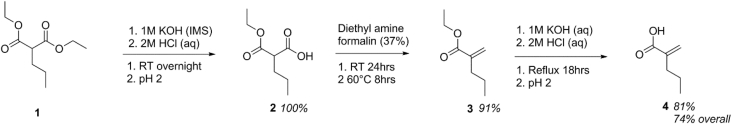
Synthesis of 2-Propyl Acrylic Acid.

**Scheme 2:**
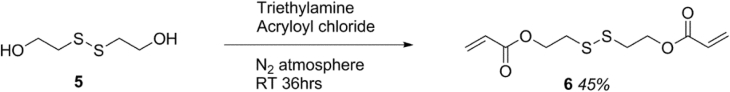
Synthesis of DSDA.

**Scheme 3:**
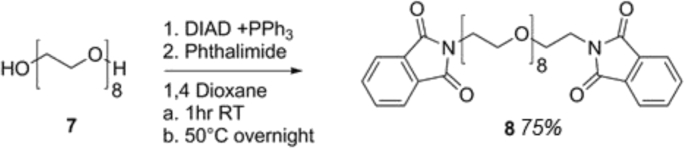
Synthesis of PEGPHT.

**Scheme 4:**
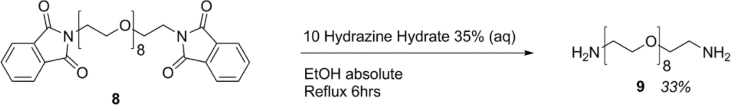
Synthesis of PEG diamine.

**Scheme 5:**
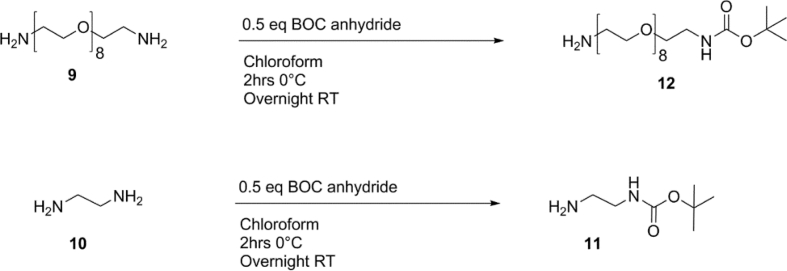
Mono Boc protection of diamines.

**Scheme 6:**
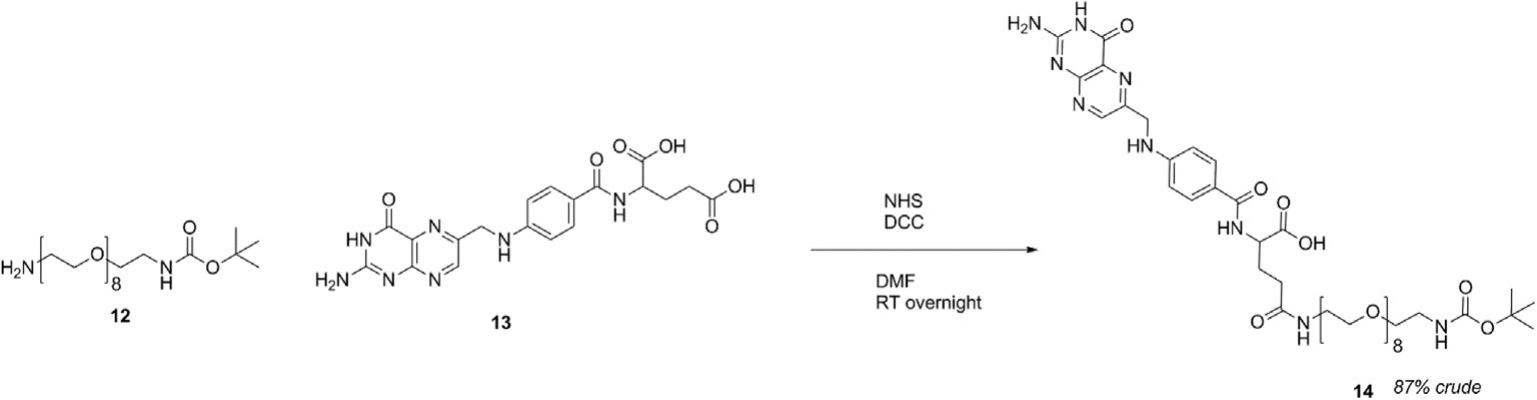
Synthesis of Boc-PEG-FOLATE.

**Scheme 7:**
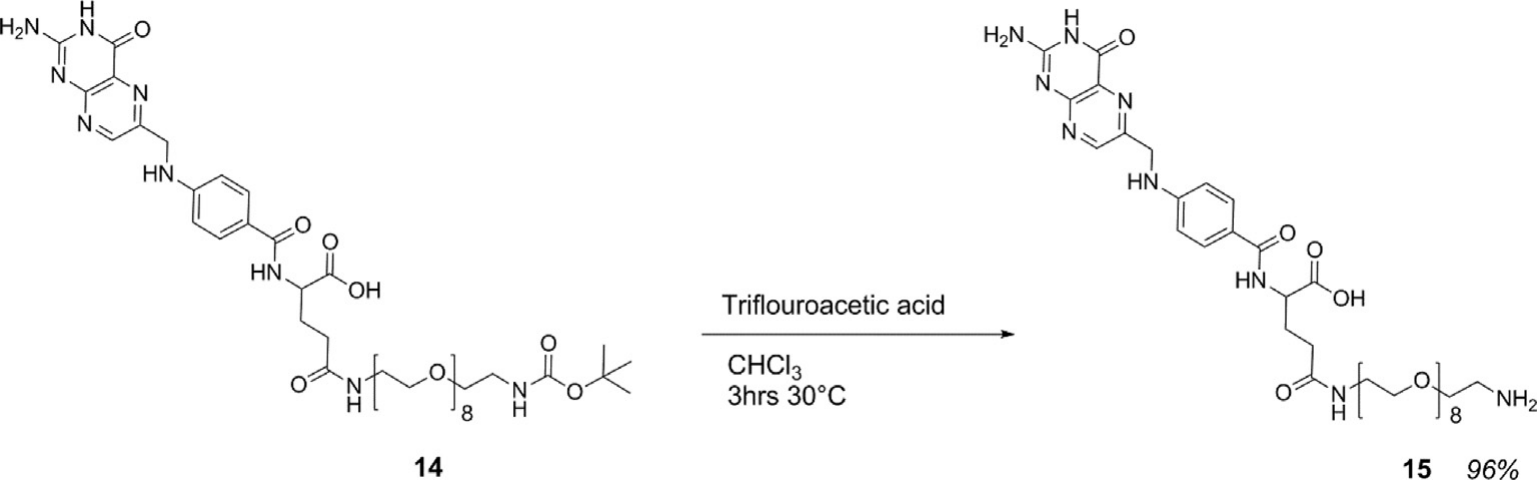
Boc deprotection to isolate NH_2_-PEG-Folate.

**Scheme 8:**
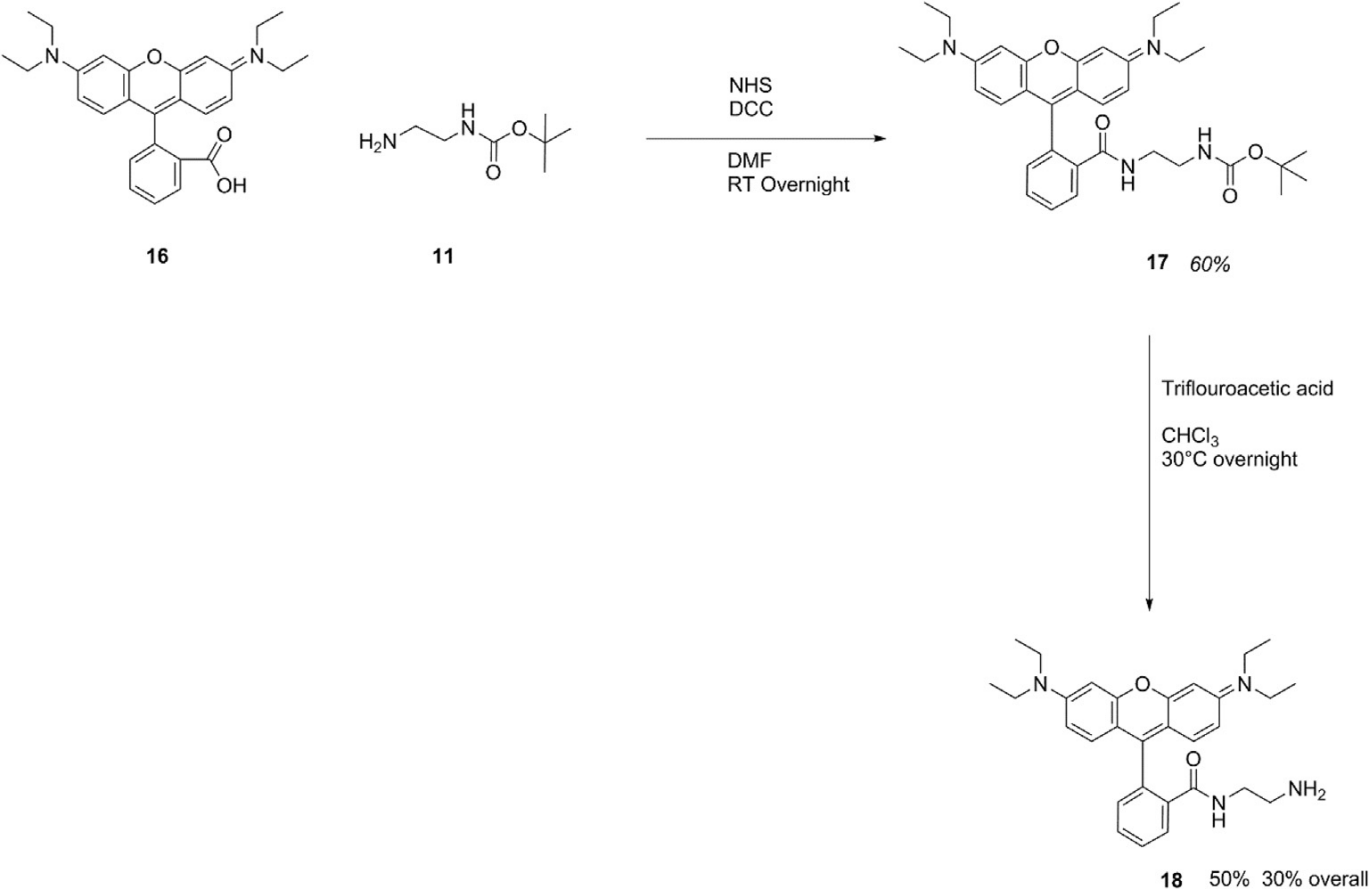
Synthesis of Rhodamine B ethylene diamine.

Data for the ^1^H NMR analysis of polymers HBP5050, HBP4060 and HBP3070 are displayed (Fig. 1, Fig. 2, Fig. 3). Size Exclusion Chromatography (SEC) traces for the synthesised polymers are presented in Fig. 4. For folate bioconjugation of HBP4060, Ultra Violet Visible (UV–Vis) spectra are presented including data from: solvent (DMF), HBP4060, HBP4060_ethyf_, HBP4060_pegf_ and folic acid (Fig. 5). RAFT agent UV–Vis spectra is presented in Fig. 6. Dynamic Light Scattering outputs are displayed for HBP4060 and HBP4060_pegf_ in aqueous media with set pH values of 7.4, 6.8 and 5.4 and in the Dulbecco's phosphate buffered saline at 0.1% (w/v) (Fig. 7, Fig. 8) with tables presenting the average particle size of each (Table 1, Table 2). In addition, ^1^H NMR spectra of HBP4060_pegf_ is displayed in Fig. 9. Furthermore experimental procedures and data pertaining to the synthesis of 2-Propyl Acrylic Acid and Disulfide Diacrylate monomers, as well as diamine linkers are presented (see Scheme 1, Scheme 2, Scheme 3, Scheme 4, Scheme 5, Scheme 6, Scheme 7, Scheme 8).

**Table 1:** Summation of Fig. 7.

Peak	pH	Particle size, nm (mean)
Purple	Dulbecco PBS	109.0
Red	7.4	100.4
Blue	6.8	74.8
Green	5.4	337.9

**Table 2:** Summation of Fig. 8.

Peak	pH	Particle size, nm (mean)
Red	Dulbecco PBS	1177.6
Blue	7.4	882.4
Green	6.8	1194.5
Purple	5.4	1160.1

## 2 Experimental design, materials and methods

### 2.1 Synthesis of monomer 2-propyl acrylic acid (PAA)[2]

Diethylpropylmalonate [1] (50 g, 0.24 mol) was added to a round bottom flask alongside 300 mL 1 M KOH solution (in IMS, Industrial Methylated Spirit) and left to stir at room temperature overnight. The solid precipitate was filtered and placed in a 1 L conical flask. The solution was then concentrated in vacuo and the resultant oil was added to the solid. The contents of the conical flask were then dissolved in a minimal volume of deionized water and acidified to pH 2 with dilute HCl, at which point an oil separated from the aqueous solution. The oil was taken up by the ether and the aqueous layer was subsequently extracted thrice with 200 mL diethyl ether. The organic phase was then dried using magnesium sulfate, filtered and concentrated to quantitatively yield 2-(ethoxycarbonyl)pentanoic acid [2] (42 g, 0.24 mol), yielding FTIR peaks at 3500–3000 and 1710 cm^−1^. The synthesised 2-(ethoxycarbonyl) pentanoic acid (42 g, 0.24 mol) was placed in a round bottom flask equipped with a reflux condenser and cooled in an ice bath before addition of diethyl amine (12.5 mL, 0.24 mol). An addition funnel was attached to the reflux condenser and charged with formalin solution (37% formaldehyde in water) (0.24 mol, 19.3 g). The contents of the addition funnel were then added dropwise to the cooled reaction vessel and subsequently allowed to warm to room temperature and allowed to stir for 24hrs. The reaction mixture was then heated to 60 °C and allowed to stir for a further 8 hrs. The reaction now consisted of two distinct layers. The mixture was again cooled with an ice bath and concentrated sulfuric acid was added until the evolution of gas was ceased and the mixture was extracted thrice with 200 mL of diethyl ether. The organic extracts were combined and dried with magnesium sulfate, filtered and concentrated in vacuo to yield crude ethyl 2-methylenepentanoate [3] (91% yield). Finally, crude ethyl 2-methylenepenatanoate (0.22 mol) was added to a round bottom flask equipped with a reflux condenser alongside 400 mL of 1 M KOH (aq) and was heated to reflux for 20hrs. The solution was then allowed to cool to room temperature and was acidified to pH 2 with dilute HCl, resulting in an oil separating from the solution. Extraction was performed 4 times with 200 mL of diethyl ether and the organics were dried with magnesium sulfate filtered and concentrated to obtain as an oil 2-propyl acrylic acid [4] (0.178mol 20g 81% yield 74% overall). ^1^H NMR (400 MHz CDCl_3_ δ) 11.67 (s, 1H COOH) 6.23 (s, 1H vinyl H) 5.58 (s, 1H vinyl H) 2.21 (t, 2H, CH_2_–CH_2_) 1.51–1.39 (m, 2H CH_3_–CH_2_) 0.87 (t, 3H, CH_2_–CH_3_).

### 2.2 Synthesis of disulfanediylbis(ethane-2,1-diyl) diacrylate (DSDA) [3]

2-2′-disulfanediylbis (ethan-1-ol) [5] (9.8 mL, 0.08 mol) was dissolved in 200 mL chloroform and placed into a 2-neck round bottom flask submerged into an ice bath whilst also being bubbled with nitrogen. Trimethylamine (44.5 mL, 0.32 mol) was added to the reaction flask and the mixture was left to stir for 20 mins. Whilst still cool and bubbling acryloyl chloride (25.8 mL, 0.32 mol) was added dropwise for 30 mins. The flask was then sealed with a nitrogen balloon and the nitrogen pipe was removed. The mixture was then left to stir for 36 hrs at room temperature. The solution was then filtered to remove solid precipitate and washed twice with 150 mL of de-ionized water and 150 mL of 0.1 M Na_2_CO_3_ (aq) solution six times before two final washes with NaCl brine. The organics were then dried with magnesium sulfate, filtered and concentrated to yield a brown oil of crude DSDA (6). Purification was afforded via column chromatography. The crude oil was passed through an aluminum oxide column washed with dichloromethane and the product fractions were concentrated to yield a dark brown oil of DSDA (Yield 45%). ^1^H NMR (400 MHz CDCl_3_) 6.54–6.27 (m, 2H 2x vinyl H) 6.18–5.95 (m, 2H 2x vinyl H) 5.79 (m, 2H 2x vinyl H) 4.45–4.28 (t, 4H 2x CH_2_–O) 2.29 (t, 4H 2x *S*–CH_2_).

### 2.3 Synthesis of PEG_8_ diamine linker [4]

10 g of PEG (7) (M_w_ 400) (25 mmol PEG, 50 mmol OH groups) was dissolved in 150 mL of 1, 4- Dioxane (anhydrous) and purged with argon for 20 minutes. Separately, a solution of Diisopropyl azodicarboxylate (DIAD) (17.7 g, 87.5 mmol, 1.75 eq) in 10 mL 1,4-dioxane (anhydrous) was added dropwise under argon to an ice cooled solution of PPh_3_ (22.95 g, 87.5 mmol, 1.75 eq) under a continuous stirring of the solution for a further 30 mins. PEG solution was then transferred under argon by use of a double-ended needle and the solution was allowed to stir for a further 30 mins at ambient temperature. As a powder, phthalimide was added (12.9 g, 87.5 mmol, 1.75 eq) and the resultant mixture was allowed to stir at ambient temperature for a further 1 hr. afterwards, the mixture was heated to 50 °C and allowed to react overnight. Extraction of the desired compound was afforded by firstly removing 1, 4-dioxane in vacuo and suspending the orange oil in 200 mL of water, washed with 50 mL ethyl acetate twice and diethyl ether once and IMS was used to break down emulsions that had formed (ca.2 mL). Extraction of the desired compound was then performed thrice upon the aqueous layer via 75 mL DCM. The latter was then dried over magnesium sulfate for 1 hr, filtered and volatiles were removed in vacuo. Precipitation of the product was then performed in ice cold diethyl ether to produce a slightly yellow oil of PEGdiPHT(8) (12.34 g, 18 mmol, 75% yield). ^1^H NMR (400 MHz CDCl_3_ δ), 7.8–7.52 (m, 4H aromatic H on phthalimide), 3.91 (t, 4H CH_2_–CH_2_-Phthalimide), 3.75 (t, 4H–CH_2_–CH_2_-Phthalimide), 3.7–3.51 (m, -[CH_2_]_2_-O).

PEGdiPHT (8) (12.34 g, 18 mmol of PEG, 36 mmol of PHT) was dissolved in 125 mL of absolute ethanol in a round bottom flask. To the flask 10 equivalents of hydrazine hydrate (35%) was added and the mixture was left to reflux for 6 hrs. The mixture was then allowed to cool and thereafter filtered to remove the white precipitate that had formed. The solution was then concentrated in vacuo and dissolved in 150 mL of DCM, filtered again and extracted with 2 mL 1 M NaOH (aq). The aqueous layer was then back extracted with 25 mL DCM and the combined organics were dried over magnesium sulfate and concentrated in vacuo. The resultant brown oil yielded PEG diamine (9), this was stored in the fridge and any free phthalamide was allowed to crystallize before being filtered, and a further precipitation into ice cold diethyl ether. (33% yield). ^1^H NMR (400 MHz CDCl_3_ δ) 3.63–3.57 (m, - [CH_2_]_2_-O), 3.48 (t, 4H–CH_2_–CH_2_–NH_2_), 2.82 (t, 4H–CH_2_–CH_2_–NH_2_).

### 2.4 Mono Boc (tert-Butyloxycarbonyl) protection of diamine derivatives [5]

For the Boc protection of ethylene diamine (10) 0.5 eq. of Boc anhydride was dissolved in 100 mL chloroform and added dropwise to an ice-cold solution of ethylene diamine over a period of 3 hrs. The mixture was stirred overnight and washed eight times with 150 mL of distilled water. The organic phase was then dried with magnesium sulfate, filtered and dried via rotary evaporation, and a clear slightly yellow oil was obtained(11). ^1^H NMR (400 MHz CDCl_3_ δ), 5.21 (s, 1H CH_2_–NH), 3.1 (s, 2H CH_2_–NH), 2.74 (s, 2H H_2_N–CH_2_), 1.36 (m, 9H BOC methyl groups), 1.12 (s, H_2_N–CH_2_) For the Boc protection of PEG_8_ Diamine, BOC anhydride (110 mg, 0.5 mmol) was dissolved in 10 mL chloroform and the mixture was added dropwise to an ice-cold solution of PEG diamine (500 mg, 1.25 mmol) over a period of 2 hrs. The mixture was stirred overnight and washed 5 times with 100 mL distilled water. The organic phase was dried under magnesium sulfate, filtered and concentrated before precipitation in cold diethyl ether to yield a brown oil 250 mg (12) (80% yield). ^1^H NMR (400 MHz CDCl_3_ δ), 3.58 (s, -[CH_2_]_2_-O), 3.46 (t, 4H –CH_2_–CH_2_–NH_2_), 2.80 (t, 4H –CH_2_–CH_2_–NH_2_), 1.37 (s, 9H BOC methyl groups).

### 2.5 Synthesis of Boc-PEG-folate

Folic acid (13) (264 mg, 0.6 mmol) was dissolved in 10 mL DMF alongside *N*-hydroxysuccinimide (115 mg, 1 mmol) and dicyclohexylurea (206 mg, 1 mmol) and left to stir at ambient temperature in the dark for 2 hrs. After this time, BOC-PEG_8_-NH2 (200 mg, 0.3 mmol) was dissolved in 1 mL of DMF and added into the reaction mixture and left to stir overnight in the dark. The mixture was then filtered, water (2 mL) was added and the product was freeze dried to remove DMF to yield 250 mg (yield 87%) crude material (14) as a brown/orange powder that was used without further purification for the next step.

### 2.6 Boc deprotection of boc-PEG-FOLATE

Boc-PEG-Folate (14) (250 mg, 0.26 mmol) was suspended in 2 mL of chloroform before the addition of trifluoroacetic acid (20 μL, 0.32 mmol) into a 10 mL reaction vial and submerged in a sand bath protected from light and left to stir at 30 °C for 3 hrs. Subsequently, the suspension had formed a dark orange solution. Volatiles were removed via rotary evaporation to yield an orange powder of NH2-PEG-Folate (15) (217 mg, 0.25 mmol, 96% yield). ^1^H NMR (400 MHz DMF-d7 δ) 10.76 (s, 1H, α-COOH) 10.50 (s, 1H, NH; guanidine on folate) 8.82 (s, 1H, N=CH) 8.71 (d, 1H, O=C–NH; folate) 8.39 (s) 8.33 (d) 7.85 (m, 2H, arH-C-C=O) 6.80 (dd, 2H, (NH-arH) 4.71–4.66 (t, 1H NH–CH) 4.66 (s, 2H, N=C–CH_2_) 3.58 (s,-[CH_2_]_2_-O), 3.48 (t, 4H –CH_2_–CH_2_–NH_2_).

### 2.7 Synthesis of Rhodamine B ethylene diamine

Mono-boc protected ethylene diamine was synthesised using the same method described in E1.4 Rhodamine B (16) (887 mg, 2 mmol) was dissolved in 10 mL DMF alongside NHS (287 mg, 2.5 mmol) DCC (516 mg, 2.5 mmol) and mono-BOC protected ethylene diamine (11) (400 mg, 2.5 mmol) and left to react overnight at ambient temperature in the dark. Solids were filtered and the resultant solution was concentrated via rotary evaporation. The resultant oil was then dissolved in 3 mL of water and then freeze dried to yield a dark red solid which was used crude (17). (700 mg, 1.2 mmol 60% yield).

The resultant compound was taken and dissolved in 4 mL DCM in a 10 mL reaction vial alongside TFA (400 μL) and submerged in a sand bath protected from light and heated to 30 °C and stirred for 2 hrs. The solution was then concentrated via rotary evaporation to yield a dark red solid (18) (291 mg, 0.6 mmol yield 50%).

### 2.8 Ninhydrin test for free amines

Polymer samples (HBP4060, HBP4060ethyf and HBP4060pegf) were dissolved in deionized water at a concentration of 4 mg/mL, whilst 2% Ninhydrin solution was dissolved in absolute ethanol. 1 mL of polymer solution and an equal amount of Ninhydrin solutions were then taken and added into a vial to make a final polymer concentration of 2 mg/mL with a final volume of 2 mL. Separately, the procedure was repeated with Glycine to produce a final concentration of 400 nmol/mL (2mL). The vials were then placed into a sand bath for 25 minutes at 100 °C to allow for the reaction to commence. The vials were then removed from the bath and allowed to cool to room temperature. Purple staining is indicative of free primary amines present (i.e. incomplete conjugation). All HBPs did not stain (negative response) whilst the glycine control for primary amine stained purple (positive response). Further characterisation was performed on a PerkinElmer Lambda 35 UV/Vis Spectrophotometer in 1 mm polystyrene cuvettes at 570 nm, however no absorbance was found from the HBP samples.

## References

[1] Blackburn C., Tai H., Salerno M., Wang X., Hartsuiker E., Wang W. (2019). Folic acid and rhodamine labelled pH responsive hyperbranched Polymers : synthesis , characterization and cell uptake studies. Eur. Polym. J..

[2] Ferritto M., Tirrell D.A. (1992).

[3] Huang Y., Sun R., Luo Q., Wang Y., Zhang K., Deng X., Zhu W., Li X., Shen Z. (2015). In situ fabrication of paclitaxel-loaded core-crosslinked micelles via thiol-ene “click” chemistry for reduction-responsive drug release. J. Polym. Sci. Part A Polym. Chem..

[4] D'Arcy R., Tirelli N. (2015). Mitsunobu reaction: a versatile tool for PEG end functionalization. Macromol. Rapid Commun..

[5] Muller D., Zeltser I., Bitan G., Gilon C. (1997). Building units for N-backbone cyclic peptides. 3. Synthesis of protected *N*^α^ -(ω-Aminoalkyl)amino acids and *N*^α^ -(ω-Carboxyalkyl)amino acids. J. Org. Chem..

